# Supplementary material for: Mapping the evolution of fertility support policies in China: A content and instrumental analysis
Source: PLoS One. 2025 Oct 9;20(10):e0332137. doi: 10.1371/journal.pone.0332137 (PMC12510515; doi:10.1371/journal.pone.0332137)
Supplement: S1 Appendix — (ZIP) [file pone.0332137.s001.zip › S1 Appendix. 226 original policy documents/51-国务院办公厅关于促进家政服务业提质扩容的意见(FBM-CLI-2-333509).doc]

国务院办公厅关于促进家政服务业提质扩容的意见

**发布部门：** [国务院办公厅](https://www.pkulaw.com/law/chl?Aggs.IssueDepartment=601) [机构沿革](javascript:void(0))

**发文字号：**国办发〔2019〕30号

**发布日期：**2019.06.16

**实施日期：**2019.06.16

**时效性：** [现行有效](https://www.pkulaw.com/law/chl?Aggs.TimelinessDic=01)

**效力级别：** [国务院规范性文件](https://www.pkulaw.com/law/chl?Aggs.EffectivenessDic=XC0203)

**法规类别：** [劳动工会综合规定](https://www.pkulaw.com/law/chl?Aggs.Category=08701) [服务](https://www.pkulaw.com/law/chl?Aggs.Category=07102)

国务院办公厅关于促进家政服务业提质扩容的意见
（国办发〔2019〕30号）

各省、自治区、直辖市人民政府，国务院各部委、各直属机构：
　　家政服务业是指以家庭为服务对象，由专业人员进入家庭成员住所提供或以固定场所集中提供对孕产妇、婴幼儿、老人、病人、残疾人等的照护以及保洁、烹饪等有偿服务，满足家庭生活照料需求的服务行业。近年来，我国家政服务业快速发展，但仍存在有效供给不足、行业发展不规范、群众满意度不高等问题。家政服务业作为新兴产业，对促进就业、精准脱贫、保障民生具有重要作用。为促进家政服务业提质扩容，实现高质量发展，经国务院同意，现提出以下意见。
　　**一、**采取综合支持措施，提高家政从业人员素质
　　（一）支持院校增设一批家政服务相关专业。原则上每个省份至少有1所本科高校和若干职业院校（含技工院校，下同）开设家政服务相关专业，扩大招生规模。开展1＋X证书制度试点，组织家政示范企业和职业院校共同编制家政服务职业技能等级标准及大纲，开发职业培训教材和职业培训包，支持家政服务相关专业学生在获得学历证书的同时，取得家政服务类职业技能等级证书。（教育部会同人力资源社会保障部、商务部等负责）
　　（二）市场导向培育一批产教融合型家政企业。到2022年，全国培育100家以上产教融合型家政企业，实现城区常住人口100万以上的地级市家政服务培训能力全覆盖。各地要以较低成本向家政企业提供闲置厂房、社区用房等作为家政服务培训基地，共享职业院校、社区教室等培训资源。（地方各级人民政府，发展改革委、教育部、商务部负责）
　　（三）政府支持一批家政企业举办职业教育。将家政服务列为职业教育校企合作优先领域，打造一批校企合作示范项目。支持符合条件的家政企业举办家政服务类职业院校，各省（区、市）要设立审批绿色通道，简化流程优化服务。推动30家以上家政示范企业、50所以上有关院校组建职业教育集团。对符合条件的家政类产教融合校企合作项目，优先纳入中央预算内投资支持范围。（教育部、发展改革委、财政部、商务部、人力资源社会保障部，地方各级人民政府负责）
　　（四）提高失业保险基金结余等支持家政培训的力度。将家政服务纳入职业技能提升行动工作范畴，并把灵活就业家政服务人员纳入培训补贴范围。所需资金按规定从失业保险基金支持职业技能提升行动专项资金中列支。地方各级政府要加大筹资力度，积极使用就业补助资金、地方人才经费和行业产业发展经费等支持家政服务培训。（人力资源社会保障部会同财政部等负责，地方各级人民政府负责）
　　（五）加大岗前培训和“回炉”培训工作力度。对新上岗家政服务人员开展岗前培训，在岗家政服务人员每两年至少得到1次“回炉”培训。组织30所左右院校和企业引进国际先进课程设计和教学管理体系。组织实施巾帼家政服务专项培训工程，开展家政培训提升行动，确保到2020年底前累计培训超过500万人次。（商务部、发展改革委、教育部、人力资源社会保障部、全国总工会、全国妇联等按职责分工负责）

　　**二、**适应转型升级要求，着力发展员工制家政企业
　　（六）员工制家政企业员工根据用工方式参加相应社会保险。大力发展员工制家政企业，员工制家政企业是指直接与消费者（客户）签订服务合同，与家政服务人员依法签订劳动合同或服务协议并缴纳社会保险费（已参加城镇职工社会保险或城乡居民社会保险均认可为缴纳社会保险费），统一安排服务人员为消费者（客户）提供服务，直接支付或代发服务人员不低于当地最低工资标准的劳动报酬，并对服务人员进行持续培训管理的企业。员工制家政企业应依法与招用的家政服务人员签订劳动合同，按月足额缴纳城镇职工社会保险费；家政服务人员不符合签订劳动合同情形的，员工制家政企业应与其签订服务协议，家政服务人员可作为灵活就业人员按规定自愿参加城镇职工社会保险或城乡居民社会保险。（人力资源社会保障部、医保局、商务部负责）
　　（七）灵活确定员工制家政服务人员工时。家政企业及用工家庭应当保障家政服务人员休息权利，具体休息或者补偿办法可结合实际协商确定，在劳动合同或家政服务协议中予以明确。（人力资源社会保障部、商务部、全国总工会，地方各级人民政府负责）
　　（八）对员工制家政企业实行企业稳岗返还和免费培训。对不裁员或少裁员的员工制家政企业按规定返还失业保险费，为符合条件的员工制家政企业员工提供免费岗前培训和“回炉”培训。（人力资源社会保障部、财政部按职责分工负责，地方各级人民政府负责）
　　（九）重点城市率先支持员工制家政企业发展。北京、上海等大中型以上城市要率先发展员工制家政企业，加大社保补贴力度，利用城市现有设施改造作为员工制家政服务人员集体宿舍。各地支持有条件的员工制家政企业提供职工集体宿舍，园区配建职工宿舍优先面向员工制家政服务人员。（相关地方人民政府负责）

　　**三、**强化财税金融支持，增加家政服务有效供给
　　（十）提高家政服务业增值税进项税额加计抵减比例。研究完善增值税加计抵减政策，进一步支持家政服务业发展。（财政部、税务总局按职责分工负责）
　　（十一）扩大员工制家政企业免征增值税的适用范围。对与消费者（客户）、服务人员签订服务协议，代发服务人员劳动报酬，对服务人员进行持续培训管理，并建立业务管理系统的家政企业提供的家政服务免征增值税。（财政部、税务总局、商务部等按职责分工负责）
　　（十二）开展家政服务“信易贷”试点。依托全国信用信息共享平台，引导和鼓励商业银行在市场化和商业自愿的前提下为信用状况良好且符合条件的家政企业提供无抵押、无担保的信用贷款。（发展改革委、人民银行、银保监会按职责分工负责，地方各级人民政府负责）
　　（十三）拓展发行专项债券等多元化融资渠道。支持符合条件的家政企业发行社会领域产业专项债券。鼓励地方运用投资、基金等组合工具，支持家政企业连锁发展和行业兼并重组。（发展改革委、商务部、人民银行、银保监会按职责分工负责，地方各级人民政府负责）

　　**四、**完善公共服务政策，改善家政服务人员从业环境
　　（十四）加强社保补贴等社会保障支持。对家政企业招用就业困难人员或毕业年度高校毕业生并缴纳社会保险费的，按规定予以社保补贴。（人力资源社会保障部，地方各级人民政府负责）
　　（十五）支持发展家政商业保险。进一步研究家政服务商业综合保险方案。鼓励家政企业参保雇主责任保险，为员工投保意外伤害保险、职业责任保险。鼓励保险公司开发专门的家政服务责任保险、意外伤害保险产品。鼓励有条件的地区组织家政企业和从业人员统一投保并进行补贴。（商务部、银保监会，地方各级人民政府负责）
　　（十六）保障家政从业人员合法权益。最大限度把家政从业人员组织到工会中，探索适合家政从业人员特点的入会形式、建会方式和工作平台。完善家政从业人员维权服务机制，保障其合法权益，促进实现体面劳动。（全国总工会负责）
　　（十七）积极推动改善家政从业人员居住条件。各地应将符合条件的家政从业人员纳入公租房保障范围，有条件的地方可集中配租，家政从业人员通过市场租房居住的，政府对符合条件的给予租赁补贴。支持各地采取多种方式帮助解决家政从业人员改善居住条件的需求。（住房城乡建设部、财政部，地方各级人民政府负责）
　　（十八）畅通家政从业人员职业发展路径。引导家政企业将员工学历、技能水平与工资收入、福利待遇、岗位晋升等挂钩。支持家政从业人员通过高职扩招专项考试、专升本等多种渠道提升学历层次。（人力资源社会保障部、教育部、商务部按职责分工负责，地方各级人民政府负责）
　　（十九）表彰激励优秀家政从业人员。五一劳动奖章、五一巾帼标兵、三八红旗手（集体）、城乡妇女岗位建功先进个人（集体）、青年文明号等评选表彰要向家政从业人员倾斜，对获得上述奖励以及在世界技能大赛和国家级一类、二类职业技能大赛中获奖的家政从业人员，纳入国家高技能人才评定范围，并在积分落户等方面给予照顾。加大家政服务业典型案例宣传力度。（商务部、人力资源社会保障部、全国总工会、全国妇联、共青团中央、发展改革委等按职责分工负责，相关地方人民政府负责）

　　**五、**健全体检服务体系，提升家政服务人员健康水平
　　（二十）分类制定家政服务人员体检项目和标准。研究制定科学合理的家政服务人员体检项目和标准，育婴员、养老护理员等职业应实行更加严格的岗前健康体检，其他从业人员上岗前应按所从事家政服务类别进行体检。（商务部会同卫生健康委负责）
　　（二十一）更好为家政服务人员提供体检服务。从事体检的医院或体检机构要明示收费标准，做好体检记录，缩短体检报告制作时间。（卫生健康委、商务部按职责分工负责，地方各级人民政府负责）

　　**六、**推动家政进社区，促进居民就近享有便捷服务
　　（二十二）支持家政企业在社区设置服务网点。家政企业在社区设置服务网点，其租赁场地不受用房性质限制，水电等费用实行居民价格。支持依托政府投资建设的城乡社区综合服务设施（场地）设立家政服务网点，有条件的地区可减免租赁费用。（商务部、住房城乡建设部、发展改革委，地方各级人民政府负责）
　　（二十三）加大社区家庭服务税费减免力度。落实好支持养老、托幼、家政等社区家庭服务业发展的税费优惠政策。（财政部、税务总局按职责分工负责）

　　**七、**加强平台建设，健全家政服务领域信用体系
　　（二十四）建立家政服务信用信息平台系统。中央财政通过服务业发展资金支持家政服务领域信用体系建设。依托全国信用信息共享平台，全量归集家政企业、从业人员、消费者的基础信息和信用信息，并按规定向相关部门及家政企业充分共享金融、税务、司法等可公开信用信息。探索建立全国家政企业和从业人员社会评价互动系统。（商务部、发展改革委、财政部按职责分工负责）
　　（二十五）优化家政服务信用信息服务。建立家政从业人员个人信用记录注册、跟踪评价和管理制度。开通家政从业人员职业背景信息验证核查渠道。依托“信用中国”网站、信用类应用程序（APP）等，按规定提供家政企业、从业人员的身份认证、信誉核查、信用报告等信息。（商务部、发展改革委、公安部按职责分工负责）
　　（二十六）加大守信联合激励和失信联合惩戒力度。基于全国信用信息共享平台建立联合奖惩系统，对在家政服务过程中存在违法违规和严重失信行为的家政企业及从业人员实行联合惩戒。开展家政企业公共信用综合评价，对信用等级较高的企业减少监管频次，提供融资、租赁、税收等便利服务。开展家政服务领域信用建设专项行动。（商务部、发展改革委按职责分工负责）

　　**八、**加强家政供需对接，拓展贫困地区人员就业渠道
　　（二十七）建立家政服务城市与贫困县稳定对接机制。把家政服务作为劳动力输出地区各类职业技能实训基地重要培训内容，在中西部人口大省重点打造一批家政服务人才培训基地。（人力资源社会保障部、商务部、全国总工会、全国妇联等按职责分工负责）
　　（二十八）建立健全特殊人群家政培养培训机制。对困难学生、失业人员、贫困劳动力等人群从事家政服务提供支持。推进“雨露计划”。为去产能失业人员、建档立卡贫困劳动力免费提供家政服务培训。（地方各级人民政府负责）

　　**九、**推进服务标准化，提升家政服务规范化水平
　　（二十九）健全家政服务标准体系。开展家政服务国家标准修订工作。完善行业标准体系。研究制定家政电商、家政教育、家政培训等新业态服务标准和规范。推进家政服务标准化试点专项行动。（商务部、市场监管总局等负责）
　　（三十）推广使用家政服务合同示范文本。规范家政服务三方权利义务关系，推广使用合同示范文本。家政企业应与消费者签订家政服务协议，公开服务项目和收费标准，明确服务内容清单和服务要求。（商务部会同人力资源社会保障部等负责）
　　（三十一）加快建立家政服务人员持证上门制度。政府通过多种方式，逐步实现统一为每一位合格的家政从业人员免费发放“居家上门服务证”，并在公共信息平台上实施诚信监管。鼓励各地参照上海等城市的先进经验，探索建立覆盖全域的家政持证上岗模式。（地方各级人民政府负责）
　　（三十二）开展家政服务质量第三方认证。家政企业要开展优质服务承诺，公开服务质量信息。开展家政服务质量监测。建立家政服务质量第三方认证制度。对家政企业开展考核评价并进行动态监管。（商务部、市场监管总局按职责分工负责）
　　（三十三）建立家政服务纠纷常态化多元化调解机制。进一步畅通“12315”互联网平台等消费者诉求渠道，发挥家政行业协会、消费者权益保护组织等作用，建立家政服务纠纷常态化多元化调解机制。（商务部、市场监管总局、全国总工会按职责分工负责）

　　**十、**发挥规范示范作用，促进家政服务业可持续发展
　　（三十四）建立健全家政服务法律法规。加强家政服务业立法研究。充分发挥家政行业协会作用，制定完善行业规范。各地要制定或者修改完善家政服务领域法规、规章、规范性文件和标准。（商务部，地方各级人民政府负责）
　　（三十五）促进家政服务业与相关产业融合发展。推动家政服务业与养老、育幼、物业、快递等服务业融合发展。大力发展家政电商、“互联网＋家政”等新业态。培育以专业设备、专用工具、智能产品研发制造为支撑的家政服务产业集群。（商务部，地方各级人民政府负责）
　　（三十六）培育家政服务品牌和龙头企业。各地要培育一批具有区域引领和示范效应的龙头企业，形成家政服务业知名品牌。实施家政服务业提质扩容“领跑者”行动。（发展改革委、商务部，地方各级人民政府负责）
　　国务院建立由发展改革委、商务部牵头的部际联席会议制度，各省级人民政府要建立家政服务工作协调机制，各地要把推动家政服务业提质扩容列入重要工作议程，构建全社会协同推进的机制，确保各项政策措施落实到位。

国务院办公厅
2019年6月16日

© 北大法宝：（[www.pkulaw.com](https://www.pkulaw.com/)) 专业提供法律信息、法学知识和法律软件领域各类解决方案。北大法宝为您提供丰富的参考资料，正式引用法规条文时请与标准文本核对。
欢迎查看所有[产品和服务](https://www.pkulaw.com/helps/2.html)。法宝快讯：[如何快速找到您需要的检索结果？](https://www.pkulaw.com/helps/70.html)[法宝](https://www.pkulaw.com/helps/69.html)V6有何新特色？

扫描二维码阅读原文

**原文链接：**<https://www.pkulaw.com/chl/fa3f0ab4b2b56a16bdfb.html>
